# Reply to: Satellite artifacts modulate FireCCILT11 global burned area

**DOI:** 10.1038/s41467-024-46169-z

**Published:** 2024-03-08

**Authors:** Adrián Cardil, Marcos Rodrigues, Renaud Barbero, Joaquin Ramírez, Cathelijne Stoof, Carlos Alberto Silva, Midhun Mohan, Pere Gelabert, Macarena Ortega, Sergio de-Miguel

**Affiliations:** 1 Joint Research Unit CTFC - AGROTECNIO - CERCA, Solsona, Spain; 2 Technosylva Inc, La Jolla, CA USA; 3 Department of Forest and Agricultural Sciences and Engineering, University of Lleida, Lleida, Spain; 4 Department of Geography and Land Management, University of Zaragoza, Zaragoza, Spain; 5 INRAE, RECOVER, Aix-Marseille University, Aix-en-Provence, 13182 France; 6 Department of Environmental Sciences, Wageningen University, PO box 47, 6700 AA Wageningen, The Netherlands; 7 Forest Biometrics and Remote Sensing Laboratory (Silva Lab), School of Forest, Fisheries, and Geomatics Sciences, University of Florida, PO Box 110410, Gainesville, FL 32611 USA; 8 Department of Geography, University of California-Berkeley, Berkeley, CA 94709 USA; 9 Forest Fire Laboratory, Department of Forest Engineering, University of Córdoba, 14071 Córdoba, Spain

**Keywords:** Climate sciences, Natural hazards

**replying to** L. Giglio & D. Roy et al. *Nature Communications* 10.1038/s41467-024-46168-0 (2024)

We acknowledge the insightful comments from Giglio and Roy on our paper entitled “Climate teleconnections modulate global burned area” published in Nature Communications (2023). After carefully reading their commentary letter, we realized that the main criticism to our work related to the dataset of burned area (BA) used in our research, namely the FireCCILT11^1^ product. They raised valid points about the quality and known limitations of that specific dataset which, despite being already addressed in the literature^2,3^, may be worth to contextualize to better framing the spatial patterns of climate teleconnection-fire domains we outlined.

We used the global BA data from the FireCCILT11 product mainly because it is the longest (1982–2018) global BA dataset freely available based on satellite imagery^2^. There is evidence that some climate teleconnections (CTs) are non-stationary, strengthening or weakening their signal on multi-decadal timescales, which may influence climate and BA^4^. Likewise, certain CTs depict signal cycles that fluctuate over long time periods (i.e. decades). Thus, the length of the time series is key when analyzing low variability CT indices such as the Atlantic Multidecadal Oscillation or the Pacific Decadal Oscillation. Therefore, encompassing the longest available period is not only advantageous, but a strong requirement to properly analyze the multiple CT-fire relationships operating at different temporal scales at the global level. Previous research did analyze the effect of AMO and PDO on BA with a time series of 18 years^5^, and we do believe that a larger time series is needed to analyze such CT patterns operating over multiple decades. Indeed, several reviewers of our original manuscript also highlighted this and encouraged us to run the analysis for the whole time series.

FireCCILT11 compiles monthly BA data estimates at 0.05 degrees cell resolution, although in our analyses data was resampled at 0.5° resolution at a monthly scale to facilitate data handling and processing while enhancing pattern recognition. BA detection was based on the LTDRAVH09 product, generated by NASA from the AVHRR 2/3 sensors using seven NOAA satellites^1^. As mentioned above, the quality of global burned area (BA) products has been already assessed in previous research^2,3^ and the limitations and assumptions on the use of global BA datasets are already known and discussed. We agree with Giglio and Roy (2023) in that these issues could have been specified more explicitly in the methods section of our manuscript, but we assumed this is an issue already discussed in the literature^6,7^. Indeed, BA datasets based on MODIS products (MCD64A1) attain a large capability to capture the BA output at moderate resolution. However, our research focused on assessing the interannual spatial-temporal association between BA and CTs. To achieve this goal, we deemed the ability to capture the temporal variability over a larger timespan more critical than potential moderate differences in BA detection. We believe that most of the available BA products would be suitable for this purpose because the temporal correlation between different products is high^2^, hence selecting the longest one available.

The purpose of our manuscript was not to evaluate the advantages and disadvantages of different global BA products, being that a matter beyond our scope. However, following Giglio and Roy’s commentary letter, exploring additional sources of BA may shed light on the potential uncertainty attributed to our procedure. Accordingly, we have rerun our analyses using an additional product (MCD64A1), and compared it with FireCCILT11, to ascertain whether or to what extent our key findings might vary depending on the product used. Analyses were replicated in the period 2001 to 2018, the longest interval with common data between both BA products within the original study period, therefore resulting in a 20-year shorter timespan compared to the published manuscript. As a sensitivity analysis, we reconstructed the BA dataset with both the FireCCILT11 and MCD64A1, then reproduced the allocation of the fire season peak (FSP) and calculated the correlation maps between CTs and BA.

The coherence between BA products resampled at the working resolution (0.5° x 0.5°) on a monthly time scale was very high (Pearson’s R = 0.92) in line with previous research^2^. Furthermore, our investigation revealed a noteworthy resemblance in the fire season peak (FSP; Fig. 1), with 73% of the global BA matching the exact FSP, and with 96% of the global BA matching the exact FSP ± 1 month. We found the best concordances in fire-prone areas such as the tropical Savanna of Africa, Australia and South America and the largest disparities in the areas where fire activity was scarce, e.g., temperate Europe, eastern United States, or certain areas within the tropical moist forest and eastern China. We also assessed the agreement between BA-CTs correlations between BA products. In regions where there was no discrepancy in the FSP, (i.e., 73% of the global burned area, as mentioned above), this association stood at 0.66 raw Pearson’s correlation coefficient (obtained from the full comparison between reported correlation coefficients for each CT and time lag), signifying a robust agreement between products. This value exhibited a downward trend as a function of the FSP difference as expected (R = 0.37 for 1-month difference and R = 0.27 with more than 1-month difference in the FSP). The latter only affected 4% of global BA.

**Fig. 1:**
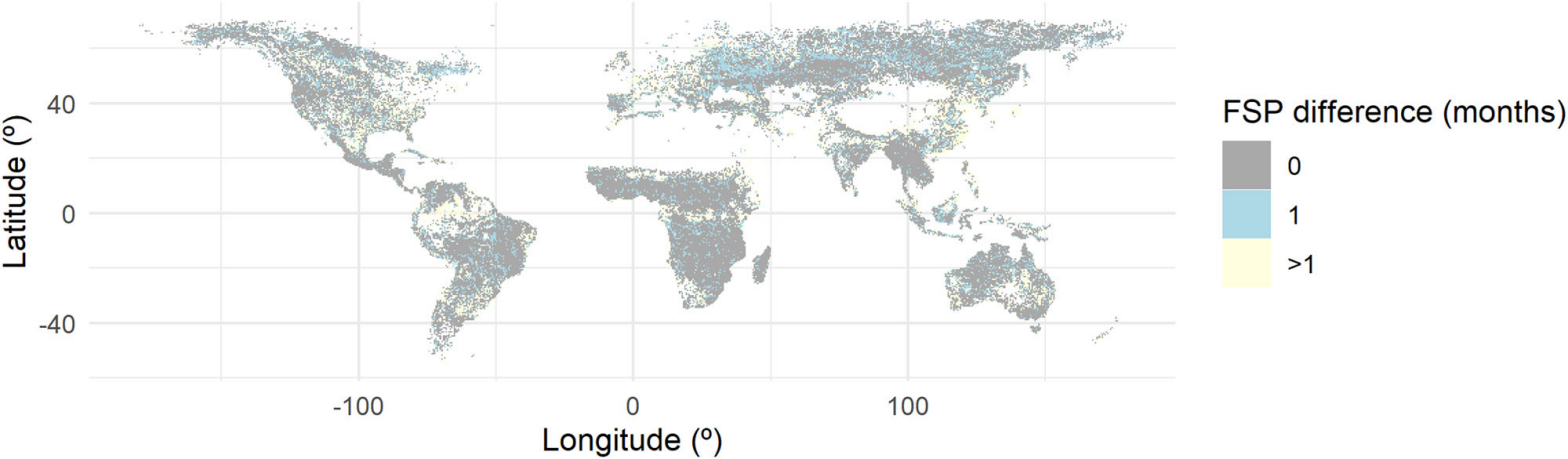
Difference in months between the fire season peak (FSP) calculated using the FireCCILT11 and MCD64A1 products from 2001 to 2018.

While fully acknowledging the inherent value, pros and cons of the several available datasets, the FireCCILT51 was considered to perform better than NASA MCD64A1 v006 as highlighted in Shi and Touge (2022)^8^ manuscript: “*Compared to the GFED 4.1* *s and GABAM datasets, Fire CCI v5.1 takes into account the characteristics of long-term and up-to-date data. Therefore, FireCCILT51 was selected as the most suitable dataset*”. Giglio and Roy stated that we incorrectly cited Shi and Touge ref. ^8^ who only used the FireCCILT51 for the 2000–2019 period and not its parent version FireCCILT11 which was used in our manuscript, a difference overlooked in our manuscript. Regardless, we hereby recognize this unintentional oversight.

Finally, we would like to emphasize that our research features a comprehensive comparison of our results against the most recent literature on the CT-Fire relationships. Extended insights into global CT-fire relationships and potential mechanisms explaining these patterns are shown in the supplementary materials of our original manuscript^9^. Our results are strongly in agreement with previous research in most regions worldwide, while contributing to unravel the contribution of specific CT patterns such as the Southern Annular Mode. Furthermore, our findings highlight the global role played by low variability modes such as the Tropical North Atlantic and the Pacific Decadal Oscillation, the reliability of which could have been questioned if a much shorter data series had been used instead.

## Data Availability

The data supporting the findings of this study are publicly available and cited within the manuscript. FireCCILT11 data is available here: https://geogra.uah.es/fire_cci/fireccilt11.php). MODIS data (MCD64A1) is available here: https://lpdaac.usgs.gov/products/mcd64a1v006/. Additional data and code related to this research are available from the corresponding author upon request.
